# Surgical treatment outcomes and risk factors for post-TB lung disease

**DOI:** 10.5588/ijtldopen.24.0322

**Published:** 2024-11-01

**Authors:** C. Guo, Q. Li, L. Wei, Y. Liu, D. Sun, C. Ding

**Affiliations:** Department of Thoracic Surgery, Xi’an Chest Hospital, Xi’an, Shaanxi, China.

**Keywords:** post-tuberculosis lung disease, PTLD, surgical treatment, risk factors, tuberculosis

## Abstract

**OBJECTIVE:**

This study aims to investigate the effectiveness and safety of surgical treatment for post-TB lung disease (PTLD) and to analyse its risk factors.

**METHODS:**

Data were collected from 268 patients who underwent pulmonary resection for TB in Xi’an Chest Hospital, Xi’an, Shaanxi, China, between January 2014 and December 2023. The efficacy and safety of the three groups were compared, and the TB group was used as the control group to analyse the risk factors of PTLD.

**RESULTS:**

The results indicated the three groups in intraoperative blood loss, post-operative drainage volume, post-operative complications, and post-operative hospital stay also varied significantly among the three groups (all *P* < 0.01). Additionally, factors such as pre-operative anti-TB therapy duration (OR = 1.02, *P* = 0.007), age (OR = 1.03, *P* = 0.030), and comorbid diabetes mellitus (OR = 3.00, *P* = 0.046) were identified as significant contributors to PTLD. While pre-operative haemoptysis demonstrates a statistically significant correlation with both precursor PTLDs, this association likely reflects the clinical expression of the underlying disease process.

**CONCLUSION:**

The study confirms that surgery for PTLD is safe and efficacious. Patients with advanced age, an extended duration of pre-operative anti-TB therapy, comorbid diabetes mellitus and pre-operative haemoptysis should maintain vigilance regarding the potential development of PTLD.

## RÉSUMÉ

**OBJECTIF:**

Cette étude vise à étudier l'efficacité et l'innocuité du traitement chirurgical de la maladie pulmonaire post-TB (PTLD, pour l’anglais « *post-TB lung disease* ») et à analyser ses facteurs de risque.

**MÉTHODES:**

Les données ont été recueillies auprès de 268 patients ayant subi une résection pulmonaire pour la TB à l'hôpital thoracique de Xi'an, Xi'an, Shaanxi, Chine, entre janvier 2014 et décembre 2023. L'efficacité et l'innocuité des trois groupes ont été comparées, et le groupe tuberculeux a été utilisé comme groupe témoin pour analyser les facteurs de risque de PTLD.

**RÉSULTATS:**

Les résultats ont indiqué que les trois groupes de perte de sang peropératoire, de volume de drainage postopératoire, de complications postopératoires et de séjour à l'hôpital postopératoire variaient également de manière significative entre les trois groupes (tous *P* < 0,01). De plus, des facteurs tels que la durée du traitement antituberculeux préopératoire (OR 1,02 ; *P* = 0,007), l'âge (OR 1,03 ; *P* = 0,030) et le diabète sucré comorbide (OR 3,00 ; *P* = 0,046) ont été identifiés comme des facteurs significatifs de la PLTD. Bien que l'hémoptysie préopératoire démontre une corrélation statistiquement significative avec les deux PTLD précurseurs, cette association reflète probablement l'expression clinique du processus pathologique sous-jacent.

**CONCLUSION:**

L'étude confirme que la chirurgie pour le PTLD est sûre et efficace. Les patients présentant un âge avancé, une durée prolongée de traitement antituberculeux préopératoire, un diabète sucré comorbide et une hémoptysie préopératoire doivent rester vigilants quant au développement potentiel d'un PTLD.

TB continues to receive widespread attention worldwide as a common respiratory infectious disease.^1^ Recent research on TB has focused chiefly on diagnosis, new drug development, and drug-resistant TB. However, despite standard anti-TB treatment leading to the disappearance of microbiological activity (conversion from positive to negative sputum culture), two-thirds of patients still exhibit significant pulmonary structural changes, manifesting as scars, fibrosis, cavities, or other alterations on imaging.^2,3^ These changes can lead to long-term respiratory symptoms, lung function impairment, and ultimately chronic respiratory diseases. Collectively, these sequelae of TB are known as post-TB lung disease (PTLD), which includes a range of conditions such as chronic obstructive pulmonary disease (COPD), bronchiectasis, aspergillosis, and organising pneumonia.^4^ The WHO’s Global Action Plan for the Prevention and Control of Noncommunicable Diseases in 2013 recognised the interaction between TB and chronic respiratory disease.^5^ However, PTLD is not mentioned in the WHO’s End TB Strategy or the Sustainable Development Goals (SDG 3.3).^6,7^ Currently, there are no guidelines or expert consensus on PTLD, necessitating further research.^8^

PTLD is a global and poorly recognised problem that has received scant attention in China. Currently, epidemiological survey data are scarce on PTLD, and research on its clinical characteristics, risk factors, prevention, and treatment remains limited.^9^ Therefore, to comprehensively evaluate the surgical outcomes of patients with PTLD and explore the factors that affect surgical outcomes, we conducted a retrospective review of data from 268 patients who underwent surgical procedures for TB at Xi’an Chest Hospital, Xi’an, Shaanxi, China. between 2014 and 2023. Of these, 71 patients were diagnosed with PTLD, 30 with precursor PTLD, and the remaining 167 patients were confirmed to have TB based on post-operative pathological findings. By analysing these risk factors, we hope to gain a deeper understanding of the pathophysiology of PTLD and identify potential areas for improvement in surgical management. The findings of this study are expected to contribute to the development of more effective surgical strategies for PTLD, ultimately leading to improved quality of life and survival rates for affected individuals.

## MATERIALS AND METHODS

### Subjects

We conducted a retrospective study on patients who underwent pulmonary resection for TB at Xi’an Chest Hospital from January 2014 to December 2023. The inclusion criteria were as follows: 1) all patients with TB met the diagnostic criteria of “WS 288-2017 Diagnosis for TB,”^10^ which includes any bacteriologically confirmed or clinically diagnosed TB, with extrapulmonary TB not excluded; 2) all confirmed cases of TB were diagnosed with at least one positive result among sputum smear, sputum Xpert^®^ MTB/RIF test (Cepheid, Sunnyvale, CA, USA), *Mycobacterium tuberculosis* (Mtb) polymerase chain reaction (PCR), Mtb RNA rapid test, tissue Mtb PCR, and Mtb culture; 3) all patients received standardised anti-TB treatment for at least 1 month, with treatment regimens conforming to medication guidelines.^11^ Surgical indications included recurrent haemoptysis or massive haemoptysis, destroyed lungs, long-term non-absorbing lesions, or persistent cavities.^12^ The exclusion criteria were 1) post-operative pathological diagnosis of malignant tumour or concurrent malignancy; and 2) pre-operative intention for pleural stripping, with intraoperative change to lung resection.

### Surgical methods

All patients underwent tracheal intubation combined with intravenous general anaesthesia, utilising a double-lumen endotracheal tube. The patient was placed in a lateral decubitus position on the healthy side during the operation for one-lung ventilation.

*Thoracoscopic surgery:* For single-port thoracoscopic surgery, an incision of 3–4 cm in length was made in the fifth intercostal space at the midaxillary line. For two-port thoracoscopic surgery, a 3–4 cm incision was made in the fifth intercostal space at the midaxillary line, and a 1 cm incision was made between the seventh intercostal space midaxillary line and posterior axillary line. For three-port thoracoscopic surgery, the incisions were made at 3 cm between the fourth intercostal space anterior axillary line and midaxillary line, 1 cm between the seventh intercostal space midaxillary line and posterior axillary line, and 1 cm between the ninth intercostal space posterior axillary line and scapular line. Lung tissue and fissures were resected using endoscopic cutting and stapling devices (ENDO-GIA; Ethicon, Raritan, NJ, USA), with blue staples for lung parenchyma, white staples for vessels, and green staples for airways.

*Open thoracotomy:* A conventional posterolateral incision of approximately 20–25 cm in length was made. For resection of the upper lobe, access was gained through the fifth intercostal space, while the sixth intercostal space was used for the lower lobe. For total lung resection, the fifth intercostal space was utilised. Lung tissue was resected using an open stapling device with blue staples, vessels were sutured with Mersilk (Medtronic, Dublin, Ireland), airways were stapled with green staples, and sharp dissection was performed. The tracheal stump of a total lung resection was additionally buried with a pollin (Ethicon) line.

### Data collection

We collected a series of indicators from medical records of patients, including age, gender, duration of pre-operative anti-TB treatment, presence of comorbid diabetes mellitus, positive sputum culture before medication, pre-operative TB resistance, duration of anti-TB drug use, intraoperative blood loss, post-operative drainage volume, post-operative chest tube removal time, post-operative complications, and post-operative hospital stay.

All selected study cases had been diagnosed with TB at the initial diagnosis, and post-operative histopathology was the sole criterion for grouping. In reality, post-operative histopathology in most cases was not a single result: it included pure tuberculous inflammation, TB complicated by necrotising pneumonia, TB with fungal infection, TB with bronchiectasis, pure organising pneumonia, pure fungal infection, organising pneumonia with bronchiectasis, bronchiectasis with fungal infection and necrosis, and so on. Cases with pure tuberculous inflammation were classified as the TB group in the grouping process. Patients whose TB is accompanied by specific pathological lung changes, such as granulomatous inflammation with caseous necrosis, fibrous thickening of the pleura, and/or bronchiectasis in the absence of active infection, are now more explicitly categorised as the precursor PTLD group. Those with post-operative histopathology showing no evidence of tuberculous changes but only inflammation, bronchiectasis, fungal infection, etc., were classified as the PTLD group. This grouping accurately reflects the histopathological changes throughout the entire process, from the initiation of TB treatment through the course of treatment to the development of PTLD after treatment completion.

### Post-operative medication and follow-up

Patients with TB must continue medication for at least 6 months post-operatively, with monthly follow-ups and computed tomography (CT) scans every 3 months until 1 year after discontinuation of the drug. Patients with drug-resistant TB must continue medication for at least 18 months after the conversion of sputum cultures to negative status postoperatively, with monthly follow-ups and bimonthly CT scans, until 1 year after the successful discontinuation of the medication.^13^ Patients with aspergillosis infection were treated with itraconazole or voriconazole for 6 months postoperatively, with monthly follow-ups and trimonthly CT scans until 1 year after discontinuation of the medication.^14,15^ For other conditions such as bronchiectasis and organising pneumonia (OP), post-operative CT scans were required every 3 months, with follow-up lasting for 6 months. Patients with TB complicated by aspergillosis infection should follow the TB follow-up protocol. The follow-up period ended in April 2024.

### Ethical approval

This study was approved by the Ethics Committee of the Xi’an Chest Hospital, Xi’an, Shaanxi, China, and was conducted according to the principles of the Declaration of Helsinki. All the participants provided written informed consents for clinical information collection and subsequent analysis at recruitment.

### Statistical analysis

The statistical analysis of the data was conducted using SPSS v25.0 software (IBM, Armonk, NY, USA). For continuous variables, descriptive statistics were performed using mean ± standard deviation (X ± SD) or median (minimum, maximum) [M (min, max)]. Intergroup comparisons were conducted using *t*-tests or non-parametric tests. χ^2^ tests were employed for categorical variables. Variables with statistically significant differences identified through univariate analysis and variables deemed influential were included in the logistic regression analysis. Odds ratios (ORs) and 95% confidence intervals (CIs) were calculated by the logistic regression model. *P* < 0.05 was considered statistically significant.

## RESULTS

A total of 268 patients were classified into three groups based on post-operative pathological findings: the TB group with 167 cases, including 104 males (62.3%) and 63 females (37.7%), with an average age of 37.80 ± 13.69 years; the precursor PTLD group with 30 cases, including 26 males (86.7%) and four females (13.3%), with an average age of 44.40 ± 12.28 years; and the PTLD group with 71 cases, including 44 males (62.0%) and 27 females (38.0%), with an average age of 44.30 ± 13.81 years. Statistically significant differences were observed among the three groups in terms of age (*P* < 0.001), sex (*P* = 0.030), initial sputum culture positive (*P* = 0.008), initial TB drug resistance (*P* = 0.016), pre-operative anti-TB treatment duration (*P* = 0.009), and pre-operative haemoptysis (*P* < 0.001). No statistical differences were observed in comorbid diabetes (*P* = 0.161) (Table 1).

**Table 1.** Basic information of three groups of patients.*

Variables		Total (*n* = 268) *n* (%)	TB (*n* = 167) *n* (%)	Precursor PTLD (*n* = 30) *n* (%)	PTLD (*n* = 71) *n* (%)	Statistic	*P* value
Age, years, mean ± SD		40.26 ± 13.89	37.80 ± 13.69	44.40 ± 12.28	44.30 ± 13.81	F = 7.27	<0.001
Sex	Female	94 (35.1)	63 (37.7)	4 (13.3)	27 (38.0)	χ^2^ = 7.01	0.030
	Male	174 (64.9)	104 (62.3)	26 (86.7)	44 (62.0)		
Comorbid diabetes	No	234 (87.3)	147 (88.0)	23 (76.7)	64 (90.1)	χ^2^ = 3.66	0.161
	Yes	34 (12.7)	20 (12.0)	7 (23.3)	7 (9.9)		
Initial sputum culture positive	No	230 (85.8)	135 (80.8)	27 (90.0)	68 (95.8)	χ^2^ = 9.62	0.008
	Yes	38 (14.2)	32 (19.2)	3 (10.0)	3 (4.2)		
Initial TB drug resistance	No	239 (89.2)	142 (85.0)	28 (93.3)	69 (97.2)	χ^2^ = 8.23	0.016
	Yes	29 (10.8)	25 (15.0)	2 (6.7)	2 (2.8)		
Pre-operative anti-TB treatment duration, months, median [IQR]		12.00 [6.50–24.00]	12.00 [6.00–23.00]	23.00 [12.00–24.00]	24.00 [9.00–36.00]	χ^2^ = 9.35	0.009
Pre-operative haemoptysis	Yes	100 (37.3)	35 (21.0)	20 (66.7)	45 (63.4)	χ^2^ = 50.77	<0.001
	No	168 (62.7)	132 (79.0)	10 (33.3)	26 (36.6)		

* Intergroup comparisons were conducted using *t*-tests or non-parametric tests. For categorical variables, χ^2^ tests were employed. *P* < 0.05 was considered statistically significant.

PTLD = post-TB lung disease; SD = standard deviation; IQR = interquartile range.

Furthermore, comparisons of surgery-related indicators revealed statistically significant differences among the three groups in intraoperative blood loss (*P* < 0.001), post-operative drainage volume (*P* = 0.003), post-operative complications (*P* = 0.001), and post-operative hospital stay (*P* = 0.019). No statistically significant difference was observed in the post-operative chest tube removal time (*P* = 0.058) (Table 2).

**Table 2.** Surgical data of all study patients.*

Variables	Total (*n* = 268) median [IQR]	TB (*n* = 167) median [IQR]	Precursor PTLD (*n* = 30) median [IQR]	PTLD (*n* = 71) median [IQR]	Statistic (χ^2^)	*P* value
Intraoperative blood loss, ml	400.00 [200.00–800.00]	300.00 [150.00–550.00]	775.00 [300.00–1,350.00]^†^	500.00 [300.00–1,000.00]^†^	22.25	<0.001
Post-operative drainage volume, ml	887.50 [575.00–1,342.50]	820.00 [450.00–1,222.50]	1112.50 [671.25–1,745.00]^†^	965.00 [747.50–1,412.50]^†^	11.85	0.003
Post-operative chest tube removal time, days	5.00 [3.00–8.00]	5.00 [3.00–7.00]	7.00 [4.25–11.75]	6.00 [4.00–9.00]	5.70	0.058
Post-operative complications, *n* (%)	61 (22.8)	26 (15.6)	10 (33.3)	25 (35.2)^†^	13.08	0.001
Bronchopleural fistula, *n*	3	2	0	1		
Hydropneumothorax, *n*	32	11	7	14		
TB dissemination, *n*	3	3	0	0		
Atelectasis, *n*	7	3	0	4		
Fat liquefaction, *n*	7	4	1	2		
Lung infection, *n*	4	1	2	1		
Heart failure, *n*	2	0	0	2		
Chronic cough, *n*	1	1	0	0		
Coagulation disorder, *n*	1	0	0	1		
Arrhythmia, *n*	1	1	0	0		
Post-operative hospital stay, months	15.00 [11.00–20.00]	14.00 [11.00–19.00]	16.50 [14.00–28.75]^†^	14.00 [11.00–20.00]	7.97	0.019

* *P* < 0.05 was considered statistically significant.

† Statistical significance compared to the TB group, and all comparisons were adjusted using Bonferroni correction.

PTLD = post-TB lung disease; SD = standard deviation; IQR = interquartile range.

Additionally, pre-operative anti-TB therapy duration (OR = 1.02, 95% CI 1.01–1.04; *P* = 0.007), age (OR = 1.03, 95% CI 1.00–1.05; *P* = 0.030), and comorbid diabetes mellitus (OR = 3.00, 95% CI 1.02–8.83; *P* = 0.046) were identified as significant influencing factors for PTLD, compared to the TB group. Pre-operative haemoptysis showed a statistically significant association with precursor PTLD (OR = 5.84, 95% CI 2.39–14.24; *P* < 0.001) and PTLD (OR = 6.49, 95% CI 3.26–12.90; *P* < 0.001), but this correlation probably mirrors the clinical manifestation of the underlying disease rather than serving as an independent risk factor (Table 3).

**Table 3.** Multinomial logistic regression analysis results.

Post-operative pathology		β	SE	Wald	OR (95%CI)*	*P* value^†^
Precursor PTLD						
Intercept		–4.471	1.319	11.493		0.001
Pre-operative anti-TB treatment duration, months		0.011	0.011	0.910	1.01 (0.99–1.03)	0.340
Age, years		0.024	0.016	2.260	1.02 (0.99–1.06)	0.133
Sex	Male				1	
	Female	–1.011	0.588	2.958	0.36 (0.12–1.15)	0.085
Pre-operative haemoptysis	No				1	
	Yes	1.764	0.455	15.044	5.84 (2.39–14.24)	<0.001
Comorbid diabetes	Yes				1	
	No	0.209	0.575	0.132	1.23 (0.40–3.80)	0.716
Initial sputum culture positive	Yes				1	
	No	0.342	0.709	0.233	1.41 (0.35–5.65)	0.629
Initial TB drug resistance	Yes				1	
	No	0.652	0.872	0.559	1.92 (0.35–10.61)	0.455
PTLD						
Intercept		–6.673	1.269	27.660		< 0.001
Pre-operative anti-TB treatment duration, months		0.022	0.008	7.303	1.02 (1.01–1.04)	0.007
Age, years		0.026	0.012	4.694	1.03 (1.00–1.05)	0.030
Sex	Male				1	
	Female	0.235	0.353	0.445	1.27 (0.63–2.53)	0.505
Pre-operative haemoptysis	No				1	
	Yes	1.870	0.351	28.443	6.49 (3.26–12.90)	<0.001
Comorbid diabetes	Yes				1	
	No	1.098	0.551	3.975	3.00 (1.02–8.83)	0.046
Initial sputum culture positive	Yes				1	
	No	1.218	0.692	3.100	3.38 (0.87–13.11)	0.078
Initial TB drug resistance	Yes				1	
	No	1.436	0.819	3.073	4.21 (0.84–20.95)	0.080

* Calculated using the logistic regression model.

† *P* < 0.05 was considered statistically significant.

SE = standard error; OR = odds ratio; CI = confidence interval; PTLD = post-TB lung disease.

## DISCUSSION

The concept of PTLD was first proposed in 2007.^16^ In a review published by Van Kampen et al. in 2018,^9^ it was noted that out of the 212 international TB guidelines retrieved, only three mentioned sequelae of TB; none of these guidelines provided information on the diagnosis, differential diagnosis, or principles of treatment for these sequelae. There are only 156 articles related to PTLD in the review, with 54 mentioning sequelae of TB, yet without specifying the types of lesions. Another 47 articles mentioned sequelae such as aspergillosis, bronchiectasis, or bronchial stenosis. These 101 articles explored the effects of surgical and respiratory support techniques on patients with PTLD. Other studies proposed that surgical treatment, medication, respiratory support techniques, modification of unhealthy behaviours (such as smoking cessation), and promotion of lung health education are effective methods.^17–18^ The authors believe that PTLD should be defined as residual or newly developed lung lesions after the treatment of pulmonary TB, leading to lung function loss, respiratory symptoms, and ultimately, evolving into chronic lung disease. The treatment of PTLD includes surgery, medication, respiratory support techniques, health education, etc., among which surgical treatment is more definitive in its effects.

From the perspective of surgical-related indicators, both the precursor PTLD group and the PTLD group exhibited a significant increase in intraoperative blood loss and post-operative drainage volume compared to the TB group. Regarding post-operative complications, the PTLD group experienced a higher incidence than the TB group. Both TB and PTLD can cause extensive structural changes in the lung parenchyma. As TB gradually progresses to PTLD, the duration of chronic inflammatory stimulation gradually increases, resulting in extensive adhesions within the chest cavity, ranging from loose membranous adhesions to dense callous-like adhesions. The mediastinal and hilar lymph nodes also gradually enlarge and may even form calcifications with unclear relationships with the surrounding tissues. Additionally, the proliferation of capillaries and the tortuosity and thickening of bronchial arteries can increase surgical difficulty, intraoperative blood loss, and post-operative drainage volume. Structural changes in lung tissue can also lead to decreased lung compliance, slow post-operative lung expansion, or even poor re-expansion. This explains why, although the incidence of post-operative complications is higher in the PTLD group than in the TB group, severe complications are uncommon in both groups, with only the incidence of post-operative hydropneumothorax being significantly higher in the PTLD group. Nonetheless, there were no deaths in either group, and post-operative follow-up results were good, indicating that while the perioperative risks may be higher in the PTLD group, the safety and efficacy of the surgical intervention are confirmed.

From the results of the logistic regression analysis, age, pre-operative anti-TB treatment duration and comorbid diabetes were identified as risk factors for developing PTLD. From a clinical perspective, the longer the duration of anti-TB treatment, the higher the likelihood of developing PTLD. This suggests that, despite successful treatment of TB with standardised anti-TB therapy, further prolongation of treatment does not necessarily lead to further improvement of lung lesions, which may have already transformed into PTLD. Diabetes, as a fundamental metabolic disorder, is inherently prone to complicate TB.^20^ While diabetes is also a risk factor for PTLD, its actual clinical significance remains debatable and may be associated with the small sample size. TB can cause structural changes in the lungs, including parenchymal lesions, airway lesions, chest wall and pleural lesions, and vascular lesions.^19^ Haemoptysis can occur when tuberculous lesions invade blood vessels. However, it is important to note that during a prolonged inflammatory process in the lungs, haemoptysis is associated not only with direct damage to the walls of blood vessels but also, to a greater extent, with an increase in pulmonary blood pressure due to the replacement of lung tissue with fibrous tissue, leading to persistent haemoptysis. As anti-TB treatment progresses, the active bacterial excretion and the presence of viable *Mycobacterium tuberculosis* diminish, indicating the disappearance of the biological characteristics of TB. Despite this, the structural damage to the lungs and vascular injury sustained during the infection is not fully repaired, and symptoms such as haemoptysis may persist due to the ongoing fibrotic changes in the lung tissue. Therefore, patients who continue to have haemoptysis after treatment for TB should be vigilant for the development of PTLD.

Research on PTLD is still relatively limited, and our retrospective analysis only provides a preliminary exploration of the risk factors for PTLD and the safety and efficacy of surgical treatment. Since it is a single-centre retrospective study, the results require further validation. Some experts overseas have already begun to conduct relevant research on PTLD,^20,21^ and there are still some questions surrounding the definition of PTLD: Can patients with a history of TB, respiratory symptoms, and changes in chest imaging all be diagnosed with PTLD? Is it necessary to include lung function impairment as one of the diagnostic criteria? Additionally, there have been reports of cases of TB complicated with lung cancer,^22^ so should TB complicated with lung cancer also be included in the category of PTLD? These questions require further discussion and consensus among experts both domestically and internationally. In our future work, we will conduct more in-depth research on PTLD.

## CONCLUSION

In summary, early diagnosis and timely treatment are essential for PTLD. Although perioperative risks may increase, surgery remains a safe and effective treatment option. However, patients with advanced age, prolonged pre-operative anti-TB treatment, comorbid diabetes, and pre-operative haemoptysis should be vigilant for the development of PTLD.
